# Tunnel Fenestration of the Mandibula after Unsuccessful Post Traumatic Treatment: A Case Report of the One Year Follow-Up

**DOI:** 10.3390/dj11020037

**Published:** 2023-02-02

**Authors:** Peter Gillner, Richard Mosch, Constantin von See

**Affiliations:** Department of CAD/CAM and Digital Technologies in Dentistry, Danube Private University, 3500 Krems, Austria

**Keywords:** periapical surgery, radicular cyst, root canal, internal granuloma, tunnel fenestration

## Abstract

Particularly severe cases with tunneled defects are rarely reported and are described only in a few case reports. This case report describes the treatment of a tunnel fenestration in the lower central jaw after unsuccessful endodontic treatment following trauma of incisors 31 and 41 over the course of six years, which led to the development of an internal granuloma and a radicular cyst in the lower jaw. The patient presented with a 2.67 cm^3^ radicular cyst displacing the surrounding tissue at regio 31 and 41, which resulted in a tunnel-like bony defect. Endodontic treatment and periapical root tip resection on teeth 31 and 41 with cystectomy, and with a 12 month follow-up, were successful in the healing of the bone defect. The preserved teeth received lithium disilicate crowns for definite restoration one year postoperatively. This treatment can be an option for the therapy of large cysts.

## 1. Introduction

Periapical lesions are the most common radiological findings on dental examination. The differential diagnoses of a periapical granuloma or a cystic process cannot be differentiated with an imaging procedure [1]. With 50–60%, radicular cysts, like the one reported in this case, represent the most common form of cysts in the odontogenic region [2,3,4]. The development of the radicular cyst is usually due to caries or an infected pulp after trauma [5]. The resulting chronic inflammation caused by bacterial endotoxins leads to the release of epithelial cell remnants of Malassez and thus to the development of the radicular cyst [5]. This is usually located apically and, in some cases, laterally on a lateral canal of the tooth root [5]. Since cyst growth is usually asymptomatic, radicular cysts are often an incidental radiological finding [5]. The affected teeth react negatively to the cold stimulus with carbonic acid snow on sensitivity and vitality. In the long term, cyst growth leads to loosening of the teeth and resorption of the surrounding bone [5]. 

Previous studies have described that healthy tissue and bone should be preserved [5,6]. Distinguishing between cystic and healthy surrounding tissue in defects with fenestration is challenging intraoperatively. For example, Gutwald et al. noted that for sufficient cystectomy, all infected tissue must be removed to avoid residuals [5]. Arotiba et al. noted that differentiation between unhealthy tissue and healthy tooth structures is impossible without histologic analysis, and therefore only partial cystectomies are useful [7]. 

Particularly severe cases with tunneled defects are rarely reported and are described in only a few case reports [8]. There is no specific guideline for their treatment. During preoperative planning, the use of bone or volume substitutes should be considered. The study by Zhao et al. stated that the use of bone substitutes does not result in a statistically significant better outcome and that the bone defect should heal only by the blood clot formed after curettage [9]. In contrast, a study by Pradeep et al. reported less resorption using the plasma rich in growth factors (PRGF) technique and bone graft substitutes over blood clotting alone [10]. To prevent further bone damage, Baumann et al. suggested a bridging plate to provide mechanical stability after cystectomy in severe cases [11].

A Partsch II cystectomy describes a complete removal of the cyst [4]. Various surgical approaches of incision techniques for performing a Partsch II cystectomy are available, such as the half-arch incision suggested by Gutwald et al., the gingival margin incision, or the trapezoidal incision at the gingival margin [5]. The esthetic result which can be accomplished following treatment, as well as how a sufficient overview of the surgical operating site can be achieved, should be considered.

In a radical cyst, the expansive growth and inflammatory response lead to devitalization of the affected tooth and may also affect the vitalization of adjacent teeth if it continues to grow [5]. To perform tooth-preserving treatment, an endodontic surgical approach is favored by Sayed et al. [12]. In contrast, Torabinejad and White demonstrated the benefits of tooth extraction followed by implant placement in their systematic review [13].

The case described here illustrates the treatment of an internal granuloma on tooth 41 and an inflamed radicular cyst in regio 31 to 41 with tunnel-like fenestrations in the anterior mandible after trauma and unsuccessful therapy.

## 2. Case Description

**Figure 1:**
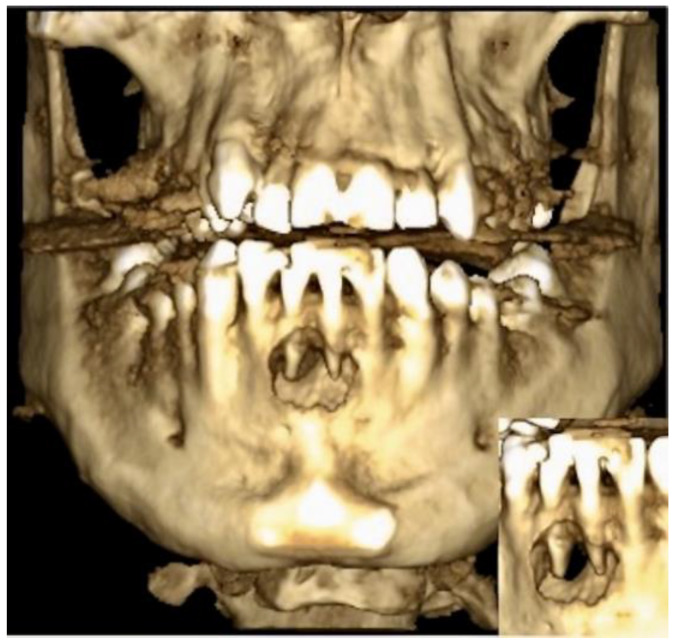
A CBCT reconstruction taken on the first appointment showing bone resorption in a tunnel-like fenestration around the central incisors of the lower jaw.

The 26-year-old male patient was presented to our dental outpatient clinic at the Department of Digital Technologies of Danube Private University in Krems for further treatment upon referral by his dentist. The patient’s general medical history was unremarkable. The patient reported blunt trauma to the anterior teeth of his mandible while playing soccer 6 years ago. The incisal edges of teeth 31 and 41 had broken off and were restored alio loco with composite restorations to restore restorative and aesthetic needs including endodontic treatment. Both teeth exhibited dark discoloration. Intraorally, there was mild swelling of the labial mucosa and an inflamed gingival margin on tooth 41, from which pus spontaneously drained. A bony defect was palpable both lingually and vestibular. The sensitivity test was negative on teeth 31 and 41, while the remaining anterior teeth 32 and 42 responded physiologically. The mandibular anterior teeth did not show increased mobility. However, teeth 31 and 41 were slightly more sensitive to percussion. Cone beam computed tomography (CBCT) (ProMax^®^ 3D Plus, Planmeca, Helsinki, Finland) was obtained for radiological evaluation. Both existing root canal fillings were found to be inadequate on the CBCT (Figure 1).

**Figure 2:**
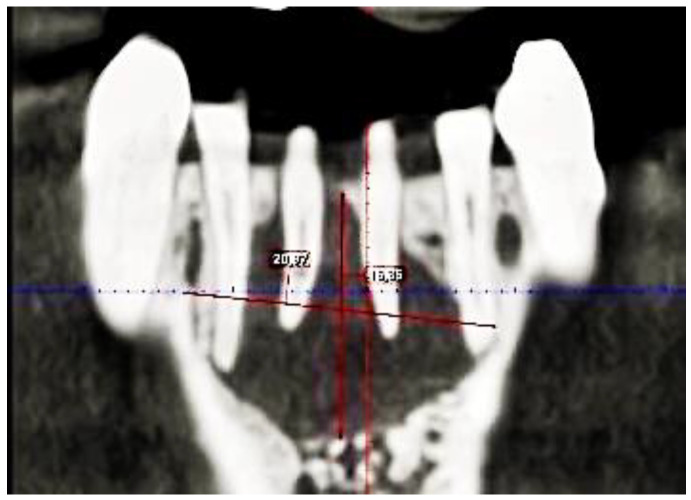
A frontal image of the CBCT taken on the first appointment showing bone resorption (with a diameter of 20.97 mm width and 16.35 mm height) of the cyst in the middle of the lower jaw.

Root perforation (via falsa) or failure to treat the second canal on tooth 41, as well as insufficient working length on tooth 31, were noted. Tooth 41 showed internal resorption in the lower third of the root with a diameter of 0.9 mm. A continuous bony ellipsoid lesion with a volume of 2.67 cm^3^ (height 16 mm, width 21 mm, and depth 8 mm) was located in the region 32 to 42 (Figure 2).

The diagnosis of a radicular cyst was accepted based on the direct location of the cyst in relation to the teeth, which had inadequate root canal treatment. Possible differential diagnoses included periapical granuloma or odontogenic keratocyst [4]. In addition, retained mesiodens 11 and 21 were found on CBCT and the patient was advised to have the maxillary third molars extracted due to their positional relationship to the second molars. After a detailed history and the patient’s consent, a combination of endodontic and surgical intervention was chosen for tooth preservation.

## 3. Clinical Procedure and Outcome

**Figure 3:**
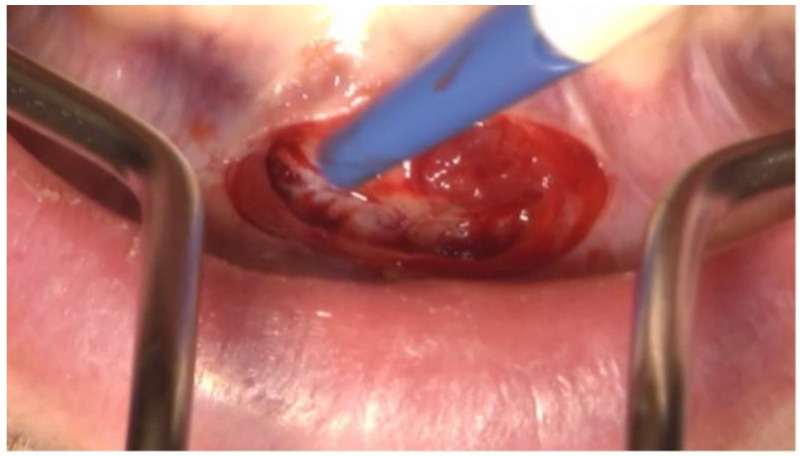
Trapezoidal incision in region 42 to 32 apical of the affected teeth for the Partsch II cystectomy one week after the first appointment.

Because of spontaneous drainage of pus, Augmentin 1000 mg (1-0-1) was prescribed preoperatively for 7 days. Intraoperatively, lingual trepanation of teeth 31 and 41 was initially performed, and orthograde root canal treatment was performed using the crown-down technique with the EndoPilot (Komet, Salzburg, Austria) up to ISO 45 diameter. Working lengths of 19 mm at 31 and 16 mm at 41 were obtained. For additional chemical disinfection, the canals were rinsed with 5 mL of sodium hypochlorite (5%) and 5 mL of aqua. The left and right mental nerves were anesthetized using 3 ampoules of Septanest lidocaine with epinephrine (Septodont 1:100,000, Niederkassel, Germany). The incision was trapezoidal, as shown in Figure 3, with the base near the gingival margin and the subsequent formation of a mucoperiosteal flap to visualize the surgical site.

**Figure 4:**
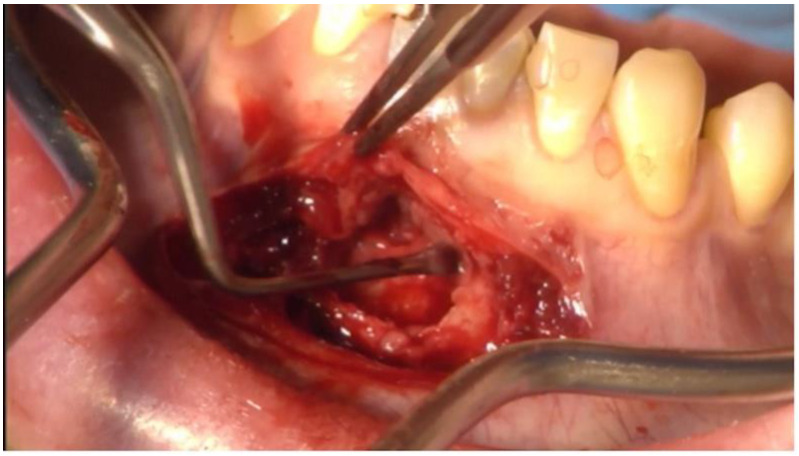
Intraoperative picture showing the dissection of the radicular cyst of the lower jaw in regio 31 to 41.

A Partsch II cystectomy was performed (Figure 4) and 3 specimens totaling 1.5 *×* 0.5 cm were obtained for histopathologic examination. After the complete removal of the cystic tissue, teeth 31 and 41 were closed in an orthograde plane under direct vision.

**Figure 5:**
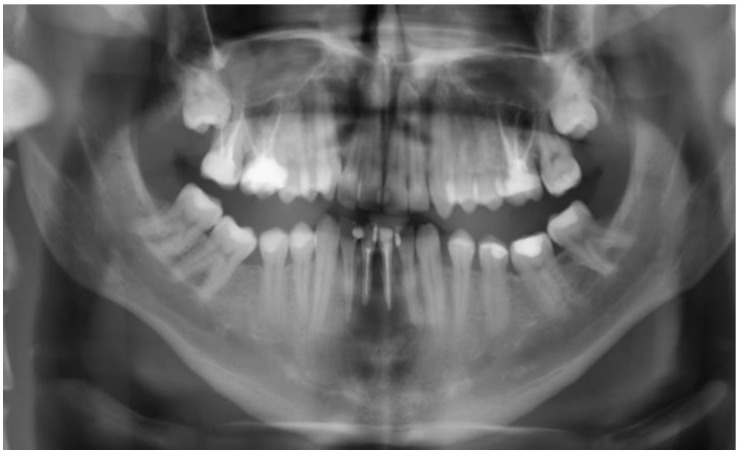
Immediate postoperative OPG showing the root canal filled teeth 31 and 41 following successful cystectomy and apicectomy.

This technique was performed with the single cone technique using ISO 45 gutta-percha points (Procodile, Komet, Salzburg, Austria) on which AH plus sealer was applied (Dentsply DeTrey GmbH, Constance, Germany). Root tip resection was performed with a rotary instrument, removing approximately 3 mm of the apical root tips at 31 and 41. Careful curettage of the surrounding bone walls was performed to ensure bleeding into the cavity and subsequent formation of blood clots. The mucosal flap was sutured deeply using two simple interrupted sutures with dissolvable sutures (PGA 4-0 PermaSharp, HU-Friedy, Frankfurt am Main, Germany) to reduce tension. The superficial wound was closed with three simple interrupted sutures (Polypropylene 5-0 PermaSharp, HU-Friedy, Frankfurt am Main, Germany). Finally, the access cavities on teeth 31 and 41 were closed with the acid-etch technique, bonding, and composite. Immediately postoperative, a panoramic radiograph (OPG), as shown in Figure 5, was obtained.

**Figure 6:**
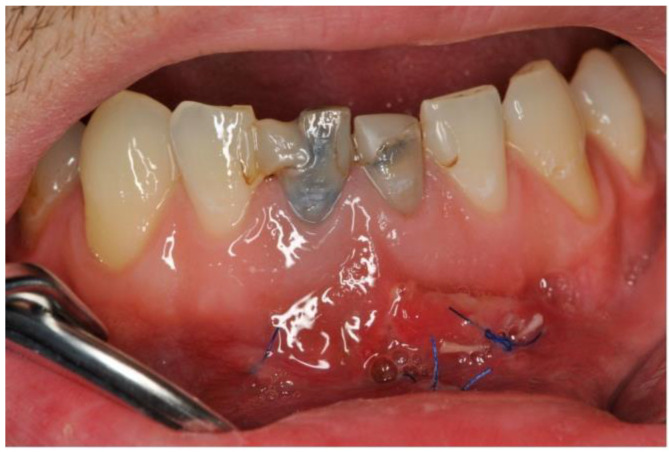
Bland wound conditions on the operation site before suture removal 13 days postoperatively.

The patient was advised to avoid strenuous physical activity for 14 days. The analgesic ibuprofen 600 mg was prescribed (max. 2400 mg/day) and antibiotic treatment with Augmentin 1000 mg (1-0-1) was prescribed for 7 consecutive days. In addition, the patient was instructed to brush the wound area carefully, and a chlorhexidine rinse was prescribed for 7 days to ensure oral hygiene. On the first day postoperatively, uniform wound healing with mild swelling of the chin and lip was observed. There was no hypoesthesia or paresthesia in the surgical area. The sutures were removed 13 days postoperatively without complications (Figure 6). The sensitivity test on teeth 32 and 42 was positive for the cold stimulus with carbonic acid snow on sensitivity and vitality.

**Figure 7:**
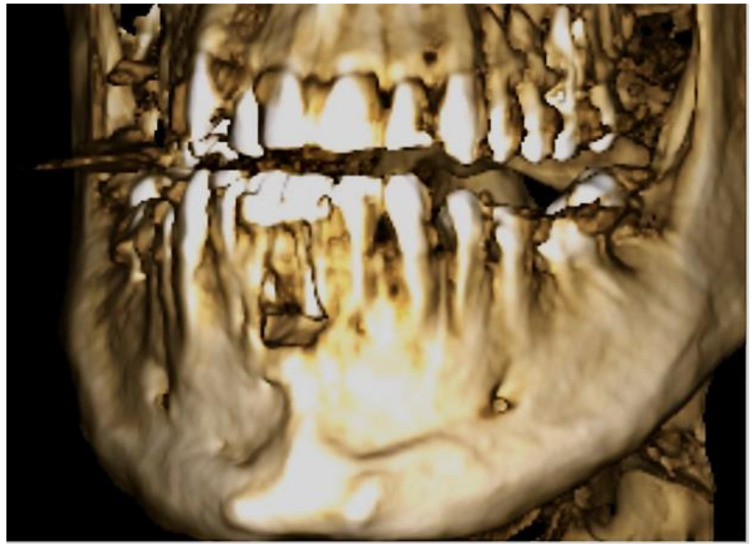
The CBCT reconstruction shows periapical healing, new bone formation, and closure of the lingual defect at the lower incisors’ region 11 months postoperatively.

A second postoperative checkup 6 weeks later revealed a self-dissolving suture protruding through the mucosa. This was shortened and the wound closed without complications. In addition, a G-ænial composite (GC Germany, Bad Homburg, Germany) was applied in a layering technique to improve esthetics in the vestibular region of 31 and 41. Histopathologic examination confirmed the tentative diagnosis of a radicular cyst. A CBCT, shown in Figure 7, was performed 11 months after surgery.

**Figure 8:**
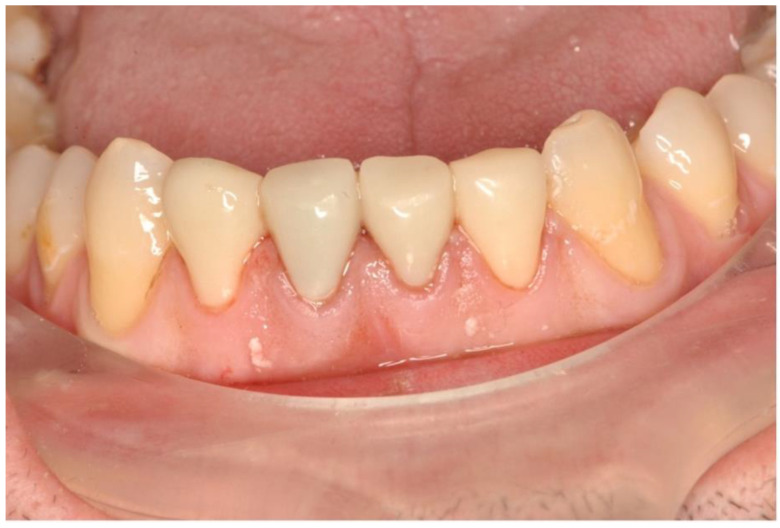
Final picture of the prosthetic rehabilitation one year after the operation with lithium disilicate crowns (E-Max, Ivoclar, Schaan, Liechtenstein) of the teeth 32 to 42.

One year after surgery, teeth 32 to 42 were restored with lithium disilicate crowns (E-Max, Ivoclar, Schaan, Liechtenstein) (Figure 8).

## 4. Discussion

With a prevalence of 25 to 30%, dental trauma is very common worldwide, with anterior teeth being the most frequently affected [14]. The goal of treatment should be to preserve the vitality of the pulp. After trauma, 10 to 15% of affected teeth develop pulp infections. If the pulp becomes non-vital, extirpation should be performed within 10 days [14]. Reevaluation should be performed clinically and radiologically at 4 weeks, 8 weeks, 6 months, and 1 year to allow assessment of progress and appropriate intervention [14,15].

The point of origin of infection into the surrounding tissue is usually apical. In rare cases, an infection may also start further laterally in the lateral canals of the root. On tooth 41 of our patient, an internal granuloma measuring 0.9 mm was found in the lower third of the root. The development of the internal granuloma can be explained by incomplete excision of the pulp [16]. This could be caused, for example, by the present Weine type IV with 2 canals and 2 foramina, which is present in 5% of mandibular central anterior teeth according to Weine et al. [16]. Another possible explanation could have been a via falsa. The final cause could not be determined at the time of surgery. The remaining pulp tissue had led to an inflammatory reaction and odontoclast stimulation. According to Thomas et al., the development of an internal granuloma is due to an inflammatory process with an incidence of about 1% [17]. Traumatic pulp necrosis of teeth 31 and 41, which had been present for years and had not been successfully treated, potentially led to the proliferation of epithelial cell remnants of Malassez. As a result of epithelial proliferation and subsequent accumulation of connective tissue, a cyst sac was formed. Cyst growth can be explained by the increase in osmolarity of the cyst fluid compared with the surrounding tissue and by the semipermeability of the membrane of the cyst sac [4,18]. The increase in size resulted in bone resorption [4]. In this case, the growth of the radicular cyst had progressed to the point where the bone had completely dissolved lingually and vestibular. This resulted in a tunnel-like fenestration.

Radicular cysts can be caused by a periodontal or apical inflammatory process. Differentiation is nearly impossible on radiographic examination. However, treatment of the cyst by cystectomy is identical to visualization, as described by Partsch [4]. The goal of a cystectomy is the complete removal of the cystic tissue. This is clinically feasible even for cysts with a bony margin, as the surgeon can safely perform enucleation through the healthy bony margin. With a piezosurgical instrument, it is possible to avoid damage to the soft tissue [19]. However, there is no comparable instrument to differentiate between healthy and cystic tissue. In this case, there was no vestibular and oral bony border and the differentiation between cyst and soft tissue at the floor of the mouth was not possible during surgery. Therefore, only partial cystectomy could be performed in this case.

Perforation of the lingual side had to be avoided to protect the blood vessels and nerves of the tongue. Therefore, curettage of the bone tissue was performed and the lingual mucoperiosteal suture was very carefully dissected along the bony defect.

The current data on the use of bone graft substitutes in these bony defects is very heterogeneous. Authors Yadav et al., Wang et al., and Fernandez et al. reported higher volume preservation and better bone quality after bone graft substitute placement [20,21,22]. It should be noted that this was often combined with the use of growth factor-rich plasma (PRGF). In contrast, the study by Taschieri et al. showed that guided tissue regeneration using inorganic bovine bone in patients with large periradicular defects had no positive effect on outcome compared with the control group [23]. The retrospective study by Rath and by Ettl et al. showed no statistical difference in the use of bone graft substitutes [5,24]. In this case, a bone graft substitute was not used due to the infected area and the associated higher risk of wound healing disorders and complications. The blood clot created by the bleeding served as an attachment point for bone regeneration.

Bridge plates are used to reconstruct bone defects in the jaw during the healing phase of the bone. In this case, there was no need for a bridging plate due to the sufficient residual bone margin and the preserved U-shape. This technique contributed significantly to successful wound healing without complications. The patient was thus spared a possible second operation and frequently occurring dehiscence [25].

Normally, a gingival incision would be preferred for a good esthetic result, which was not used in this case due to the purulent drainage of the pocket on tooth 41. The alternative of a half-arch incision was also rejected. In the event of subsequent implant placement and wound closure, the scar would be located in the esthetic zone. Thus, due to the large extent of the cyst and to additionally obtain a good overview of the surgical field, a trapezoidal incision was chosen with the base close to the gingival margin just below the cyst. In this way, the emergence profile and papillae could also be preserved for a better esthetic result [26].

The decision to preserve the mandibular anterior teeth was made despite the given circumstances, which would have been a reason for extraction, because of the high success rate for apicoectomies reported in the literature. Depending on the study, the success rate 5 years after an apicoectomy ranged from 57 to 78% [24,27,28]. In this case, a high probability of success can be assumed due to the root anatomy and good accessibility of the mandibular teeth. In addition, the patient’s age argued for tooth preservation rather than implant placement. The systematic review by Tomasi et al. defined a prognosis for tooth loss between 1 and 3.5% over 10 to 30 years, while the rate for implant loss was between 1 and 18% over 10 years [29].

At follow-up, the wound healed without irritation, and the patient had little postoperative pain. Follow-up radiographs showed adequate healing of the bone defect.

## 5. Conclusions

Therapy of the radicular cyst by orthograde root canal filling with simultaneous apicoectomy on the affected nonvital teeth and cystectomy resulted in healing of the bone defect. The retained teeth received the final prosthetic restorations after successful healing.

## Data Availability

Not applicable.
